# A case of “smoldering” immune‐mediated thrombotic thrombocytopenic purpura manifesting as recurrent cardioembolic stroke

**DOI:** 10.1002/ccr3.4850

**Published:** 2021-10-12

**Authors:** Mark E. Pepin, Eyad Saca, Soo Y. Kwon, Jori May

**Affiliations:** ^1^ Department of Biomedical Engineering University of Alabama at Birmingham Birmingham AL USA; ^2^ Department of Neurology University of Alabama at Birmingham Birmingham AL USA; ^3^ Department of Medicine Division of Hematology/Oncology University of Alabama at Birmingham Birmingham AL USA

**Keywords:** adalimumab, microangiopathy, thromboembolic stroke, thrombotic thrombocytopenic purpura

## Abstract

Prompt recognition and treatment for thrombotic thrombocytopenic purpura (TTP) are critical to prevent the irreversible manifestations of this rare and quickly fatal hematologic disorder. Untreated TTP is typically a rapid‐onset disease with mortality exceeding 90% within days in the absence of appropriate treatment. In the current report, we describe a case of immune‐mediated TTP (iTTP) in a 62‐year‐old man manifesting as longstanding thrombocytopenia, recurrent cardioembolic strokes, and valvular thrombogenesis over a period of 3 years. We provide correlative evidence to support the potential contribution of adalimumab, a TNFα inhibitor, to the development of iTTP. We offer several educational insights regarding the identification of atypical presentations of iTTP owing to the longstanding disease course and numerous clinical comorbidities seen in this patient.

## Graphical abstract

We report an atypical manifestation of immune‐mediated thrombotic thrombocytopenic purpura (TTP) that we hypothesize represents a longstanding, or “smoldering,” process. As typified by this case, the prompt recognition and treatment for immune‐mediated TTP are often confounded by clinical comorbidities. Thus, TTP should be considered in cases of unremitting thrombocytopenia of unknown origin in patients with stroke or other thrombosis.

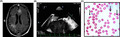

## 1 INTRODUCTION

Thrombotic thrombocytopenic purpura (TTP) can be an immune‐mediated (iTTP), or more rarely an inherited deficiency of a disintegrin and metalloproteinase with a thrombospondin type 1 motif, member 13 (ADAMTS13).^1^, ^2^ ADAMTS13 is responsible for the proteolytic cleavage of von Willebrand factor (vWf) into inactive multimeric fragments, mitigating the spontaneous aggregation of platelets and resultant microvascular thrombosis.^3^ Thus, decreased ADAMTS13 activity in TTP results in thrombotic microangiopathy with a classic pentad of thrombocytopenia, hemolytic anemia, acute kidney injury, fever, and neuropsychiatric sequelae.^4^ Without prompt treatment via plasma exchange, iTTP is rapidly fatal in up to 90% of patients.^5^

## 2 CASE PRESENTATION

We report the case of a 62‐year‐old man who presented to our emergency department with acutely altered mental status and right‐sided weakness after he was found down by a family member. He had two prior hospital admissions within the 2 years prior to presentation for acute ischemic stroke, as well as two emergency department visits for suspected transient ischemic attacks (TIAs). At the time of his first stroke (right middle cerebral artery (MCA) occlusion), a large mitral valve vegetation was identified, requiring bioprosthetic mitral valve replacement due to suspicion for endocarditis. Following his second stroke (multifocal ischemia in the right MCA distribution), he began treatment with apixaban (Eliquis®) owing to concern for recurrent cardioembolic stroke, and he continued to take this until the time of his current presentation. Records also revealed a history of plaque psoriasis, for which he was given adalimumab (Humira®) for the last 3 years, initiated approximately 1 year prior to his initial stroke.

On admission, he was afebrile and hemodynamically stable. Physical examination revealed an alert man with severe expressive and receptive aphasia, and a harsh left apical diastolic murmur. Laboratory evaluation revealed a hemoglobin of 9.4 g/dl, platelet count of 58,200/mm^3^, and creatinine of 1.7 mg/dl (consistent with known chronic kidney disease).

**FIGURE 1:**
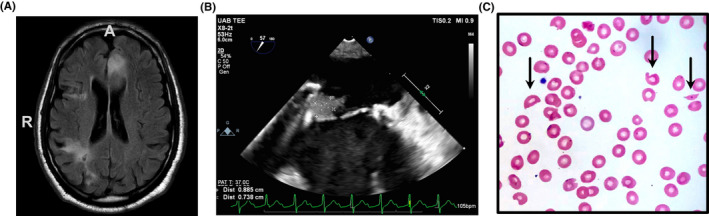
(A) Axial T2 FLAIR magnetic resonance imaging demonstrating the acute left ACA distribution infarction and multiple prior infarctions. (B) Two‐dimensional trans‐esophageal echocardiography of prosthetic mitral valve demonstrating a 1.5 cm vegetation. (C) Peripheral blood smear prior to initiation of plasma exchange therapy

Computed tomography (CT) angiogram revealed a left anterior cerebral artery (ACA) occlusion, and magnetic resonance imaging (MRI) confirmed an acute ischemic stroke in the left ACA territory. The MRI also showed multiple prior infarcts (Figure 1A **)**. Two‐dimensional trans‐thoracic echocardiogram showed echogenic material adjacent to the anterior mitral leaflet of his bioprosthetic mitral valve; a follow‐up trans‐esophageal echocardiogram demonstrated a 1.3 × 0.7cm multilobed mass attached to the atrial side of the bioprosthetic leaflet (Figure 1B). Given concern for bacterial endocarditis, empiric antibiotics were started. However, extensive infectious workup in the ensuing days was altogether unrevealing. Meanwhile, his thrombocytopenia had worsened, decreasing to 30,900/mm^3^ by the third inpatient day.

To evaluate causes of his thrombocytopenia, an extensive autoimmune workup was performed along with peripheral blood smear (Figure 1C), which revealed greater than 6 schistocytes per high power field. ADAMTS13 activity was obtained at this time, measuring <1%. All other autoimmune laboratory studies resulted negative, including ANA, lupus anticoagulant, anticardiolipin (IgG/IgM), anti‐β2‐glycoprotein I (IgG/IgM), c‐ANCA, p‐ANCA, anti‐SSA, anti‐SSB, and rheumatoid factor.

Therapeutic plasma exchange therapy (PEX) was promptly initiated for the treatment of TTP, at which time the ADAMTS13 inhibitor titer was also measured. Although initially equivocal, repeat testing was diagnostic for iTTP with a titer of >8.0 Bethesda Units.

**FIGURE 2:**
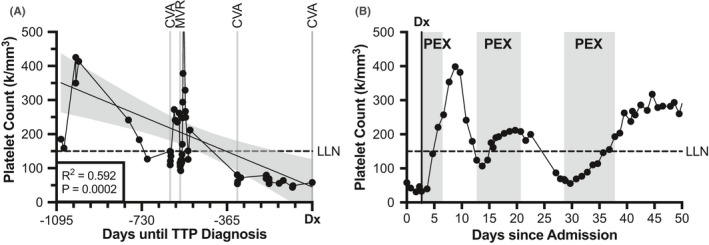
Longstanding negative correlation between platelet decline and thromboembolic complications. (A) Three‐year trend of decreasing platelet levels since initiation of adalimumab (adal.), noting 3 cerebrovascular accidents (CVA) and prosthetic mitral valve replacement (MVR). Simple linear regression (black line) with 95% confidence interval (gray shading) were performed. (B) Platelet levels plotted throughout admission, noting diagnosis with iTTP (Dx) and three intervals of therapeutic plasma exchange (PEX). For both graphs, the lower limit of normal (LLN) platelet levels are shown

In the search for additional explanations of the patient's recurrent valvular pathology, pathologic evaluation of his original mitral valve vegetation was reviewed. The report described a fibrin thrombus with focal underlying fibrotic mitral valve tissue, a finding more descriptive of an aseptic thrombus than a bacterial vegetation. Review of his records also revealed a gradual, 3‐year decline in his platelet count (*ρ* = ‐0.592, *p* < 0.001) (Figure 2A), a pattern interrupted only by the multiple transfusions received during mitral valve replacement.

The platelet count recovered to >150,000/mm^3^ after three daily treatments with PEX along with 80 mg prednisone daily (Figure 2B). The patient exhibited symptomatic improvement on PEX, with alertness and cognition returning to his pre‐admission baseline. However, the patient relapsed shortly after PEX cessation, with drop in platelet count, rise in LDH, and regression of ADAMTS13 to undetectable values. PEX was again initiated, along with rituximab, and the patient achieved remission. In the subsequent year, the patient had multiple relapses of iTTP confirmed by decline in platelet count and recurrence of undetectable ADAMTS13 with an elevated inhibitor titer, receiving treatment with caplacizumab, vincristine, and ultimately splenectomy. His disease was in remission at the time of manuscript composition, approximately 1 year after his iTTP diagnosis. He has had no further thromboembolic events since the initiation of treatment for iTTP.

## 3 DISCUSSION

Immune‐mediated thrombotic thrombocytopenic purpura is classically a rapidly fatal disease without prompt recognition and treatment. Herein, we report a potentially under‐recognized, “smoldering” variant of iTTP presenting with chronic, progressive thrombocytopenia and recurrent thromboembolic stroke.

Although an ADAMTS13 level was not checked during the patient's preceding multi‐year period of thrombocytopenia and thromboembolic stroke, the fact that his platelet count normalized for the first time since his initial stroke with initiation of PEX suggests a “smoldering” form of iTTP was present in the years prior (Figure 2A). If the thrombocytopenia had been related to medication (such as adalimumab) or another condition, improvement with PEX would not be expected. Furthermore, although we cannot definitively prove that the mitral valve thrombus and was related to iTTP in the absence of ADAMTS13 testing at diagnosis, this possibility is supported by the fact that the patient had recurrent thromboembolic stroke despite appropriate anticoagulation, and has since experienced resolution of thromboembolic events for 1 year since initiation of iTTP‐directed therapy. Although rare, previous cases of iTTP with endocarditis have been reported.^6^ Importantly, alternative causes of non‐infective endocarditis including systemic lupus erythematosus and antiphospholipid syndrome were excluded. The patient's thrombocytopenia was largely unrecognized during two previous stroke events, raising the question of whether prompt identification, with peripheral smear evaluation to identify schistocytes, could have resulted in earlier iTTP diagnosis and the avoidance of additional thromboembolic events.

While the precise trigger for this patient's iTTP cannot be verified, the timing of his platelet count decline and adalimumab initiation for treatment of plaque psoriasis suggests this medication may have contributed (Figure 2A). Adalimumab has been reported to cause thrombocytopenia via other mechanisms,^7^, ^8^, ^9^ though its role in acquired ADAMTS13 deficiency due to TNFα inhibition has not yet been reported. Nevertheless, our patient's adalimumab was suspended following iTTP diagnosis out of concern as a potential to trigger a relapse.

## 4 CONCLUSION

We report a case of apparent “smoldering” iTTP which manifested as chronic thrombocytopenia and recurrent cardioembolic strokes in the setting of recurrent valvular thrombogenesis that resolved with iTTP‐directed therapy. We provide correlative evidence to support TNFα inhibition as a yet‐unrecognized trigger of iTTP. Lastly, the current case underscores the importance of a systematic clinical approach to obtain both prompt recognition and treatment for iTTP, even for atypical presentations.

## CONFLICTS OF INTEREST

None declared.

## AUTHOR CONTRIBUTIONS

All authors contributed to the clinical diagnosis and treatment for the patient presented in this case. M.E.P. analyzed laboratory data, created figures, and drafted the case report. All authors reviewed and edited the report for accuracy and clarity. J.M. provided critical oversight regarding the interpretation and revision of the report. As such, J.M. is the guarantor of this work and accepts responsibility for its integrity.

## 5 ETHICAL APPROVAL

In accordance with the Health Insurance Portability and Accountability Act of 1996, all laboratory values, images, and patient information are presented in a manner that preserves the patient's confidentiality. Owing to the patient's poor condition, his next‐of‐kin provided consent for the publication of his case.

## CONSENT

Published with written consent of the patient.

## Data Availability

The data that support the findings of this study are available on request from the corresponding author. The data are not publicly available due to privacy or ethical restrictions.
